# Quantitative X-ray microradiography for high-throughput phenotyping of osteoarthritis in mice

**DOI:** 10.1016/j.joca.2014.04.015

**Published:** 2014-10

**Authors:** J.A. Waung, S.A. Maynard, S. Gopal, A. Gogakos, J.G. Logan, G.R. Williams, J.H.D. Bassett

**Affiliations:** Molecular Endocrinology Group, Department of Medicine, Imperial College London, London W12 0NN, UK

**Keywords:** Osteoarthritis, Mouse phenotyping, Subchondral bone, Bone mineral content, Digital X-ray microradiography, Destabilisation of the medial meniscus

## Abstract

**Objective:**

To investigate and validate digital X-ray microradiography as a novel, high-throughput and cost-effective screening approach to identify abnormal joint phenotypes in mice.

**Method:**

Digital X-ray microradiography was used to quantify the subchondral bone mineral content (BMC) in the medial tibial plateau. Accuracy and reproducibility of the method were determined in 22 samples from C57BL/6(B6Brd;B6Dnk;B6N-Tyr^c-Brd^) wild-type mice. The method was then validated in wild-type mice that had undergone surgical destabilisation of medial meniscus (DMM) and in a genetically modified mouse strain with an established increase in trabecular bone mass.

**Results:**

The measurement of subchondral BMC by digital X-ray microradiography had a coefficient of variation of 3.6%. Digital X-ray microradiography was able to demonstrate significantly increased subchondral BMC in the medial tibial plateau of male mice 4 and 8 weeks after DMM surgery and in female mice 8 weeks after surgery. Furthermore, digital X-ray microradiography also detected the increase in subchondral BMC in a genetically modified mouse strain with high trabecular bone mass.

**Conclusion:**

Quantitation of subchondral BMC by digital X-ray microradiography is a rapid, sensitive and cost-effective method to identify abnormal joint phenotypes in mice of both genders at several ages.

## Introduction

Osteoarthritis is the commonest joint disorder; it causes inexorable joint dysfunction, pain and disability as no drugs are available to prevent or delay disease progression. Patients are asymptomatic in the early stages of osteoarthritis and develop problems only after significant cartilage erosion has occurred. The aetiology of osteoarthritis is complex and multi-factorial with major genetic and environmental contributions^1^. Its heritability is estimated to be between 40 and 65% but currently identified disease susceptibility loci account for only a fraction of this heritability^2^. Thus, there is an urgent need to advance understanding of the pathogenesis of osteoarthritis and define new molecular pathways that facilitate the development of novel treatments.

Animal models are essential for *in vivo* study of disease mechanisms, drug targets and treatment responses^3^. A limited number of mouse models of spontaneous cartilage disorders have been described, including the STR/ort and STR-1N mouse. These models reinforce the importance of genetics in disease susceptibility but their use is limited by variable disease penetrance^4^. Surgically induced models of osteoarthritis are also used to investigate disease pathogenesis and response to treatment. However, this approach requires significant expertise and experience, and requires large numbers of mice at considerable expense. The best characterised and most reliable surgical model requires destabilisation of the medial meniscus (DMM), resulting in a high incidence of osteoarthritis in male mice 8 weeks following surgery^5^. Nevertheless, there is now a clear need to develop robust and rapid screening approaches to identify joint abnormalities in mouse models of spontaneous osteoarthritis.

The International Knockout Mouse Consortium and Medical Research Council *N*-ethyl-*N*-nitrosourea mutagenesis programmes aim to disrupt each of the >20,000 protein-coding genes^6^. These initiatives provide a unique opportunity to identify novel genetic determinants and *in vivo* models of osteoarthritis and define the cellular and molecular basis of disease. Nevertheless, to capitalise on these resources and identify mice with outlier joint phenotypes, new high-throughput, sensitive, specific and cost-effective joint phenotyping methods are needed.

Established osteoarthritis is characterised by articular cartilage destruction and subchondral bone sclerosis. Articular cartilage degradation progresses from surface fibrillation to formation of fissures and erosions, and finally cartilage loss with exposure of underlying bone. These changes are associated with increased chondrocyte hypertrophy, cartilage calcification and osteophyte formation^1^. Subchondral bone distributes load-bearing compressive forces and comprises the cortical plate underlying the articular cartilage and the adjacent trabecular bone. Early osteoarthritis is characterised by thinning of the cortical plate due to accelerated remodelling, whereas subchondral sclerosis is the hallmark of established disease^7^.

The gold standard histological assessment of articular cartilage type, architecture and integrity in accordance with Osteoarthritis Research Society International (OARSI) recommendations^4^ is laborious, expensive and unsuitable for high-throughput screening. Micro-computed tomography (CT) analysis of osteoarthritis in mice is also time consuming; suitable high-resolution imaging equipment is expensive and not widely available and the vast quantity of data generated requires an extensive archive^8^.

We previously established that quantitative digital X-ray microradiography is a sensitive and specific method for determining bone mineral content (BMC)^9^ and also demonstrated its suitability for high-throughput phenotyping^10^. Thus, we generated reference data for BMC in C57BL/6(B6Brd;B6Dnk;B6N-Tyr^c-Brd^) wild-type mice and subsequently screened 100 knockout strains from the IKMC to identify nine outlier strains with either high or low BMC^10^. Since subchondral bone sclerosis is a consistent and established feature of both human osteoarthritis and surgically induced mouse models of osteoarthritis^11^, ^12^, analysis of subchondral BMC represents a suitable target for high-throughput joint phenotype screening approaches.

We hypothesised, therefore, that digital X-ray microradiography can be used to quantify subchondral BMC and identify osteoarthritis phenotypes in genetically modified mice. We, therefore, studied knee joints from mice that had undergone DMM surgery^5^, ^13^ and genetically modified mice with increased trabecular bone mass^14^, ^15^.

## Methods

### Animals

Studies were conducted in accordance with the Animals (Scientific Procedures) Act 1986 and recommendations of the Weatherall report, and were approved by the Imperial College Ethical Review Committee. All wild-type mice used for DMM surgery were of the same C57BL/6(B6Brd;B6Dnk;B6N-Tyr^c-Brd^) genetic background^10^. Additional studies were performed using TRα^0/0^ and control mice originally derived in a mixed C57BL/6 and 129Sv genetic background^14^, ^15^, and subsequently backcrossed onto C57BL/6N for more than 20 generations.

### DMM surgery

Transection of the right medial meniscotibial ligament was performed at 10 weeks of age and sham surgery was performed on the left knee^5^. Males were sacrificed 4 weeks (*n* = 6 per group) or 8 weeks (*n* = 12 per group) post-surgery. Females were sacrificed 8 weeks post-surgery (*n* = 6 per group).

### Sample preparation

**Fig. 1:**
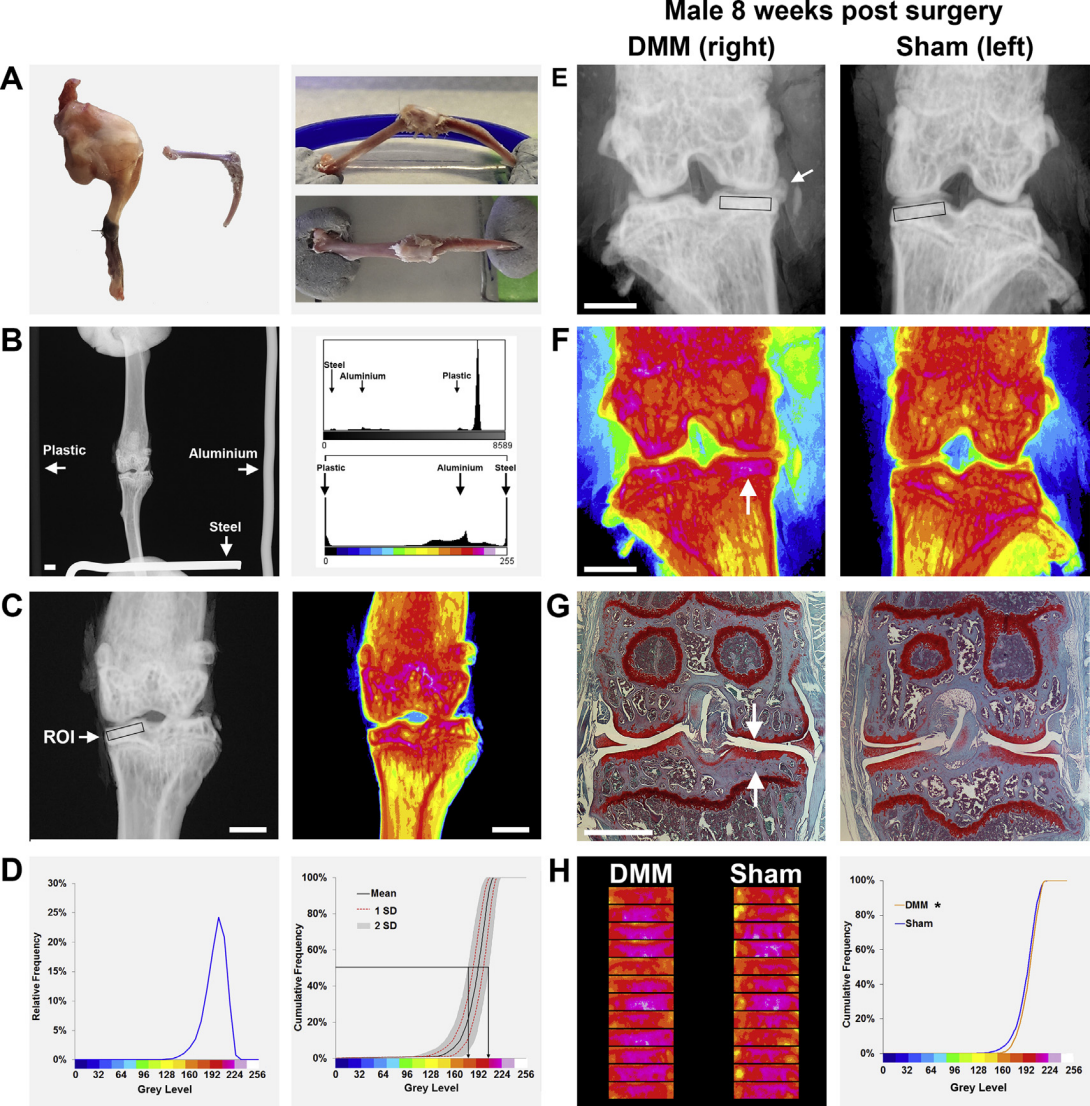
**Quantitative digital X-ray microradiography of subchondral bone**. (A) Left panel shows mouse lower limb before and after removal of muscle to expose the articulated femur and tibia with preservation of the knee joint capsule and the patella. Right panels show lateral and anterior–posterior views of the knee fixed such that the tibial growth plate lies in a vertical plane. (B) Left panel shows a Faxitron MX-20 X-ray microradiograph illustrating the essential requirement to orientate the knee joint with the patella in the mid-sagittal plane. The recommended organisation of plastic, aluminium and steel calibration standards is also shown. White bar = 1000 μm. The upper right panel shows a histogram of grey levels derived from the original 16 bit DICOM X-ray microradiograph with the location of the calibration standards indicated. The large peak on the right represents the background. The lower right panel shows the histogram after stretching the grey scale distribution to the plastic and steel standards, converting to an 8 bit TIFF format and pseudo-colouring the resultant image using a 16 colour look-up table. (C) Left panel shows a grey scale image of the left knee joint with the medial tibial plateau subchondral bone ROI (1000 μm × 250 μm box, ROI) indicated. Right panel shows a pseudo-coloured TIFF image of the same grey scale image in which lower BMC is green and yellow and higher BMC is red and purple. White bar = 1000 μm. (D) Left panel shows a representative example of a relative frequency histogram of grey levels within the medial tibial plateau subchondral bone ROI. Right panel shows the cumulative frequency histogram of relative tibial plateau subchondral BMC in *n* = 22 female, 16-week-old wild-type mice (mean ± 2.0SD reference interval in grey, ±1.0SD red dotted lines). Median grey levels at the limits of the 2.0SD reference interval are shown. (E) Grey scale images of the right (8 weeks after surgical DMM) left knee (following sham surgery) joints. The white arrow indicates medial displacement of the medial meniscus following DMM surgery. Boxes indicate the medial tibial plateaux subchondral bone ROIs. White bar = 1000 μm. (F) Corresponding pseudo-coloured images in which lower BMC is green and yellow and higher BMC red and purple. The white arrow indicates increased subchondral BMC in the medial tibial plateau following DMM surgery. White bar = 1000 μm. (G) Coronal sections through the knee joint at the level of the tibial insertion of the anterior cruciate ligament stained with safranin O and fast green. Upper arrow indicates a defect in the articular cartilage surface of the medial tibial plateau, and the lower arrow indicates increased subchondral bone following DMM surgery. White bar = 1000 μm. (H) Left panel shows a montage of the medial tibial plateaux ROIs from male mice 8 weeks following DMM (*n* = 12) or sham (*n* = 12) surgery. Right panel shows the cumulative frequency histograms of tibial plateau subchondral BMC following DMM or sham surgery. Kolmogorov–Smirnov test, DMM vs sham, *P < 0.05.

Lower limb samples were fixed in 10% neutral buffered formalin overnight and stored in 70% ethanol. Soft tissue was removed but integrity of the knee capsule and patella was preserved [Fig. 1(A)].

### Histological analysis

Knees were decalcified in 10% Ethylenediaminetetraacetic acid (EDTA) for 10 days and embedded in paraffin. 5 μm coronal sections were cut at 80 μm intervals at 16 levels (10 sections per level) through the entire thickness of the joint, stained with safranin O and fast green, according to OARSI recommendations^4^. Qualitative analysis was performed to confirm the expected presence of cartilage degradation that co-localised with subchondral bone thickening in the medial tibial plateau following DMM surgery^11^.

### X-ray microradiography

Knee joints were held in a flexed position of 105° using synthetic rubber adhesive within a 37 mm diameter plastic ring. This resulted in consistent location of the apex of the knee joint 12 mm above the mount with the tibial growth plate in a vertical plane. The patella was used as a landmark to ensure correct orientation of the joint [Fig. 1(B)].

Anterior–posterior projection X-ray images were recorded at 10 μm pixel resolution using a Faxitron MX-20 variable kV point projection X-ray source and digital image system (Qados, Cross Technologies plc, Berkshire, UK) operating at 26 kV for 15 s and 5× magnification. Images were calibrated by X-raying a digital micrometer. The relative medial tibial plateau subchondral BMC was determined by comparison with standards included in each image (1 mm diameter steel, aluminium and polyester wires) [Fig. 1(B)]. The 2368 × 2340 16 bit DICOM images were converted to 8 bit TIFF using ImageJ (http://rsb.info.nih.gov/ij/) and the grey scale histogram was stretched from the polyester (grey level 0) to steel (grey level 255) standards. Increasing gradations of mineralization density were represented in 16 equal intervals by a pseudo-colour scheme^9^, ^10^.

### Image analysis

A 1000 μm × 250 μm area of the medial tibial plateau was selected as representative of subchondral bone and defined as the region of interest (ROI) [Fig. 1(C)]. A single montage was generated from the individual ROIs from all mice within one experimental group. The relative and cumulative frequency distributions of grey levels were determined and compared between groups as indicated in the figures^9^, ^10^ [Fig. 1(D)].

### Statistics

As grey level frequency distributions were not normally distributed [Fig. 1(D)], the median value was determined for each sample. To define the variation in median BMC within the population of 22 female, 16-week-old wild-type mice [Fig. 1(D)] the mean and standard deviation of the median BMC values were determined. The Kolmogorov–Smirnov test was used to compare grey level cumulative frequency distributions between DMM and sham-operated mice, and between TRα^0/0^ and control mice. *P* values for the *D* statistic were *D* ≥ 6.01, *P* < 0.05; *D* ≥ 7.20, *P* < 0.01; and *D* ≥ 8.62, *P* < 0.001^9^.

## Results

To determine the accuracy and reproducibility of digital X-ray microradiography, we analysed knee joints from 22 female wild-type C57BL/6(B6Brd;B6Dnk;B6N-Tyr^c-Brd^) mice. The 2.0SD reference interval for relative subchondral BMC was 178–206 with a median grey level of 192 and coefficient of variation (CV) of 3.6% [Fig. 1(D)].

To validate this novel imaging approach, DMM was selected as an established surgical model of osteoarthritis^5^, ^13^ and histology was performed according to OARSI recommendations^4^ and analysed qualitatively. X-ray microradiography of joints from male mice 8 weeks following DMM surgery confirmed displacement of the medial meniscus and demonstrated increased medial tibial plateau subchondral BMC [Fig. 1(E), (F)]. Importantly these changes co-localised with articular cartilage lesions and histological evidence of increased subchondral bone [Fig. 1(G)]. Quantitative ROI analysis demonstrated a significant increase in medial tibial plateau subchondral BMC 8 weeks following DMM surgery compared to the contralateral sham-operated knee [Fig. 1(H)].

**Fig. 2:**
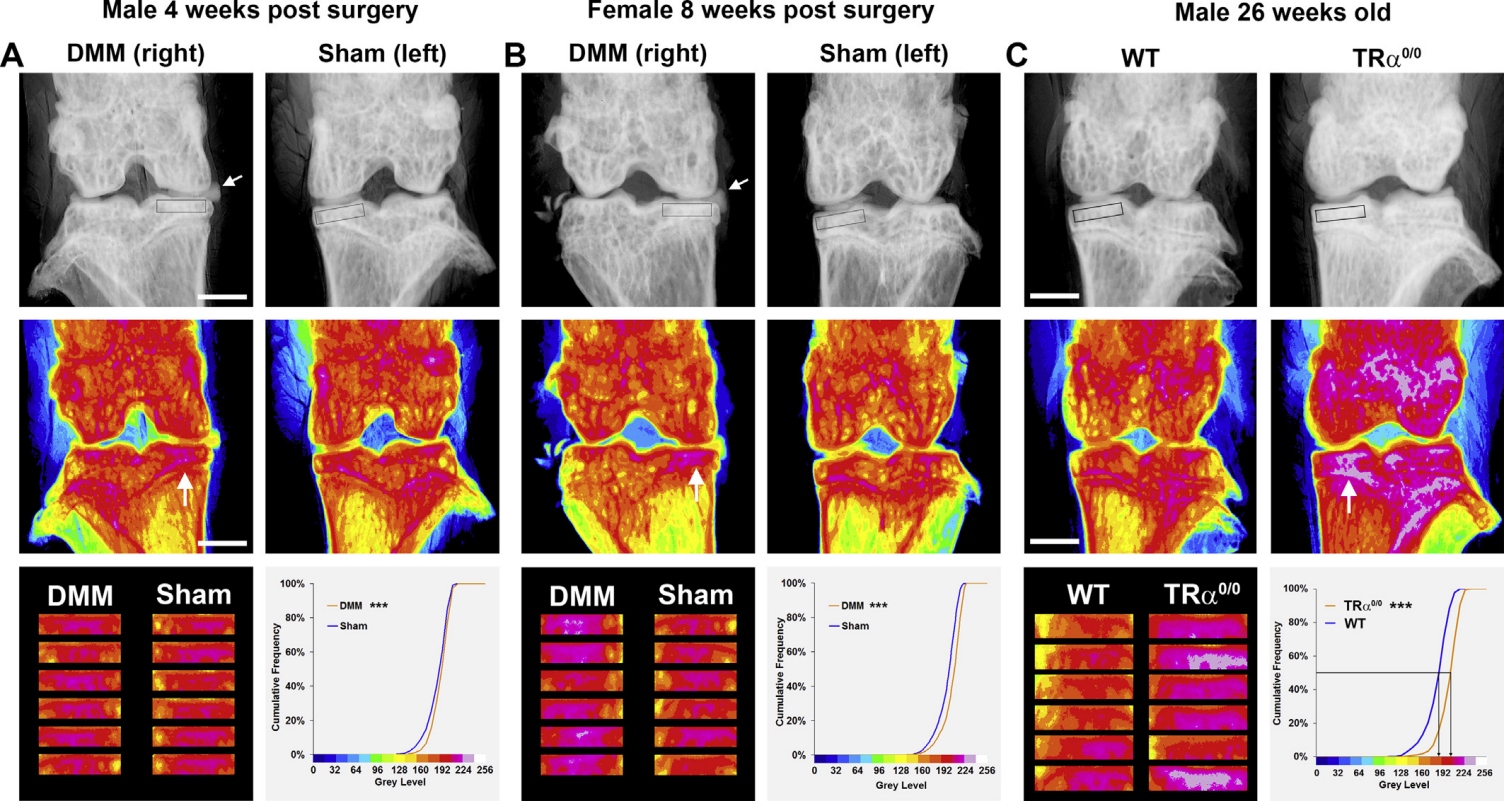
**Increased subchondral BMC in male and female mice following DMM surgery and in TRα^0/0^ mice.** (A) Upper panels show grey scale images of the right and left knees 4 weeks after DMM or sham surgery in male mice. The white arrow indicates displacement of the medial meniscus following DMM surgery. Boxes indicate subchondral bone ROIs. Middle panels show corresponding pseudo-coloured images. The white arrow indicates increased subchondral BMC following DMM surgery. Lower left panel shows the ROI montage from male mice 4 weeks following DMM or sham surgery (*n* = 6 per group). Lower right panel shows the cumulative frequency histograms of tibial plateau subchondral BMC following DMM or sham surgery. Kolmogorov–Smirnov test, DMM vs sham, ***P < 0.001. White bar = 1000 μm. (B) Upper panels show grey scale images of the right and left knees 8 weeks after DMM or sham surgery in female mice. The white arrow indicates displacement of the medial meniscus following DMM surgery. Boxes indicate subchondral bone ROIs. Middle panels show corresponding pseudo-coloured images. The white arrow indicates increased subchondral BMC following DMM surgery. Lower left panel shows the ROI montage from female mice 8 weeks following DMM or sham surgery (*n* = 6 per group). Lower right panel shows the cumulative frequency histograms of tibial plateau subchondral BMC following DMM or sham surgery. Kolmogorov–Smirnov test, DMM vs sham, ***P < 0.001. White bar = 1000 μm. (C) Upper panels show grey scale images of the left knee from 26-week-old male wild-type and TRα^0/0^ mice. Boxes indicate subchondral bone ROIs. Middle panels show corresponding pseudo-coloured images. The white arrow indicates increased subchondral BMC in TRα^0/0^ mice. Lower left panel shows the ROI montage from wild-type and TRα^0/0^ mice (*n* = 6 per group). Lower right panel shows the cumulative frequency histograms of tibial plateau subchondral BMC with the ROI median grey level in wild-type and TRα^0/0^ mice indicated. Kolmogorov–Smirnov test, TRα^0/0^ vs wild type, ***P < 0.001. White bar = 1000 μm.

To investigate sensitivity of the method, knees were analysed from males 4 weeks following DMM surgery and from females 8 weeks post-surgery [Fig. 2(A), (B)]. Medial tibial plateau subchondral BMC was increased 4 weeks post-surgery in males (*P* < 0.001) and also in females 8 weeks post-surgery (*P* < 0.001) compared to the sham-operated knee.

To determine whether the method could be applied for rapid detection of altered subchondral BMC in genetically modified mice, we analysed knees from control and TRα^0/0^ mice^15^, which we previously demonstrated exhibit increased trabecular bone mass^14^ [Fig. 2(C)]. Quantitative X-ray microradiography identified the marked increase in medial tibial plateau subchondral BMC in TRα^0/0^ mice compared to control mice (median grey level 210 vs 192; TRα^0/0^ vs control; *P* < 0.001).

## Discussion

Osteoarthritis is a major cause of pain and disability, its pathogenesis is poorly defined, and no drugs are available that prevent the onset or progression of disease. Advancement of the understanding of disease pathogenesis has been limited by a lack of suitable mouse models, but the generation of knockout mice for all protein-coding genes^6^ now provides a timely opportunity to identify new *in vivo* models of osteoarthritis. These models will be essential to investigate the cellular and molecular basis of osteoarthritis and identify novel signalling pathways for future therapeutic targeting.

Nevertheless, identification of knockout strains with outlier joint phenotypes requires development of novel, robust, high-throughput and cost-effective screening approaches since current methods are too labour intensive, time consuming and costly.

We have previously shown that X-ray microradiography is an ideal high-throughput bone phenotyping method for determination of BMC in knockout mice^9^, ^10^. We, thus, investigated this approach for the quantitation of subchondral BMC, which is characteristically increased in established osteoarthritis^7^. Induction of osteoarthritis by transection of the right medial meniscotibial ligament^5^, ^13^ was used to validate the use of X-ray microradiography for identification of osteoarthritis phenotypes in mice. Establishment of a normal reference range for medial tibial plateau subchondral BMC revealed a CV of only 3.6%, which is similar to the CV of 1.7% previously determined for whole femur BMC^10^, thus demonstrating excellent sensitivity of the method.

Digital X-ray microradiography clearly demonstrated increased subchondral BMC following DMM surgery, and these changes co-localised with the characteristic histological abnormalities in mice following surgically induced osteoarthritis^5^, ^13^. Group sizes of only six animals were sufficient to identify increased subchondral BMC 4 weeks after DMM surgery in males and 8 weeks post-surgery in females (in which provocation of osteoarthritis is less penetrant^5^). Comparing groups of six samples also allowed the difference in subchondral BMC in TRα^0/0^ mice compared to genetically identical controls to be identified. Importantly, these group sizes are commensurate with the number of samples available from knockout lines generated for the International Mouse Phenotyping Consortium^6^.

These data indicate that X-ray microradiography is a sensitive and robust technique for quantitation of subchondral BMC, but a number of technical points need to be considered. Firstly, all soft tissue should be removed whilst carefully maintaining the knee joint capsule intact. Secondly, joints should be mounted at a constant angle of flexion such that the tibial growth plate is in a vertical plane with the patella central. Thirdly, X-ray intensity and exposure should be optimised for each imaging system to ensure the full dynamic range of the detector is used. Finally, standards should be included within every image for internal calibration^9^, ^10^.

In conclusion, these studies demonstrate that quantitation of subchondral BMC by digital X-ray microradiography is a rapid new method to identify abnormal joint phenotypes in mice. It should be used as an initial high-throughput screening tool to identify mutant mice for further investigation. Such detailed studies, however, are costly and labour intensive, requiring high-resolution micro-CT, surgical provocation and gold standard OARSI histological scoring. Thus, the role of this novel method is in the cost-effective and robust identification of mouse strains with joint abnormalities that merit detailed analysis^4^, ^11^.

Overall, we report the novel application of digital X-ray microradiography that, in combination with complementary methods of joint analysis, will help to identify new genetic determinants and animal models for osteoarthritis.

## Author contributions

GRW (graham.williams@imperial.ac.uk) and JHDB (d.bassett@imperial.ac.uk) take responsibility for the integrity of the work as a whole from its inception to the finished article. JAW acquired, assembled, analysed and interpreted data, and drafted the article. SAM acquired and analysed data, and helped draft the article. SG acquired and analysed data, and helped draft the article. AG analysed and interpreted data, and helped draft the article. JGL interpreted data and helped draft the article. GRW conceived and designed the studies, provided statistical advice, interpreted data, and drafted and critically revised the article for important intellectual content. JHDB conceived and designed the studies, provided statistical advice, analysed and interpreted data, prepared final figures and critically revised the article. All authors approved the final article. JAW, GRW and JHDB obtained funding from 10.13039/501100000341Arthritis Research UK, the 10.13039/100004440Wellcome Trust and 10.13039/501100000761Imperial College London.

## Conflict of interest

JAW receives grant support from Arthritis Research UK, GRW and JHDB receive grant support from Arthritis Research UK and the Wellcome Trust. There are no other competing interests for any of the authors.

## Role of the funding source

JAW was funded by an Arthritis Research UK Clinical Research Fellowship. SAM and SG were funded by an Imperial College London MRes/PhD Programme. AG and JGL were funded by a Wellcome Trust research grant (101123/Z/13/A). The funding bodies had no role in the study design, collection, analysis and interpretation of data, writing of the manuscript, or in the decision to submit the manuscript for publication.
